# Toward dynamic, anisotropic, high-resolution, and functional measurement in the brain extracellular space

**DOI:** 10.1117/1.NPh.9.3.032210

**Published:** 2022-05-11

**Authors:** Xueqi Xu, Xiaoqian Ge, Hejian Xiong, Zhenpeng Qin

**Affiliations:** a University of Texas at Dallas, Department of Mechanical Engineering, Richardson, Texas, United States; b University of Texas at Dallas, Department of Bioengineering, Richardson, Texas, United States; c University of Texas at Southwestern Medical Center, Department of Surgery, Richardson, Texas, United States; d University of Texas at Dallas, The Center for Advanced Pain Studies, Richardson, Texas, United States

**Keywords:** optical imaging, diffusion, extracellular space, brain

## Abstract

Diffusion of substances in the brain extracellular space (ECS) is important for extrasynaptic communication, extracellular ionic homeostasis, drug delivery, and metabolic waste clearance. However, substance diffusion is largely constrained by the geometry of brain ECS and the extracellular matrix. Investigating the diffusion properties of substances not only reveals the structural information of the brain ECS but also advances the understanding of intercellular signaling of brain cells. Among different techniques for substance diffusion measurement, the optical imaging method is sensitive and straightforward for measuring the dynamics and distribution of fluorescent molecules or sensors and has been used for molecular diffusion measurement in the brain. We mainly discuss recent advances of optical imaging-enabled measurements toward dynamic, anisotropic, high-resolution, and functional aspects of the brain ECS diffusion within the last 5 to 10 years. These developments are made possible by advanced imaging, such as light-sheet microscopy and single-particle tracking in tissue, and new fluorescent biosensors for neurotransmitters. We envision future efforts to map the ECS diffusivity across the brain under healthy and diseased conditions to guide the therapeutic delivery and better understand neurochemical transmissions that are relevant to physiological signaling and functions in brain circuits.

## 1 Introduction

**Fig. 1:**
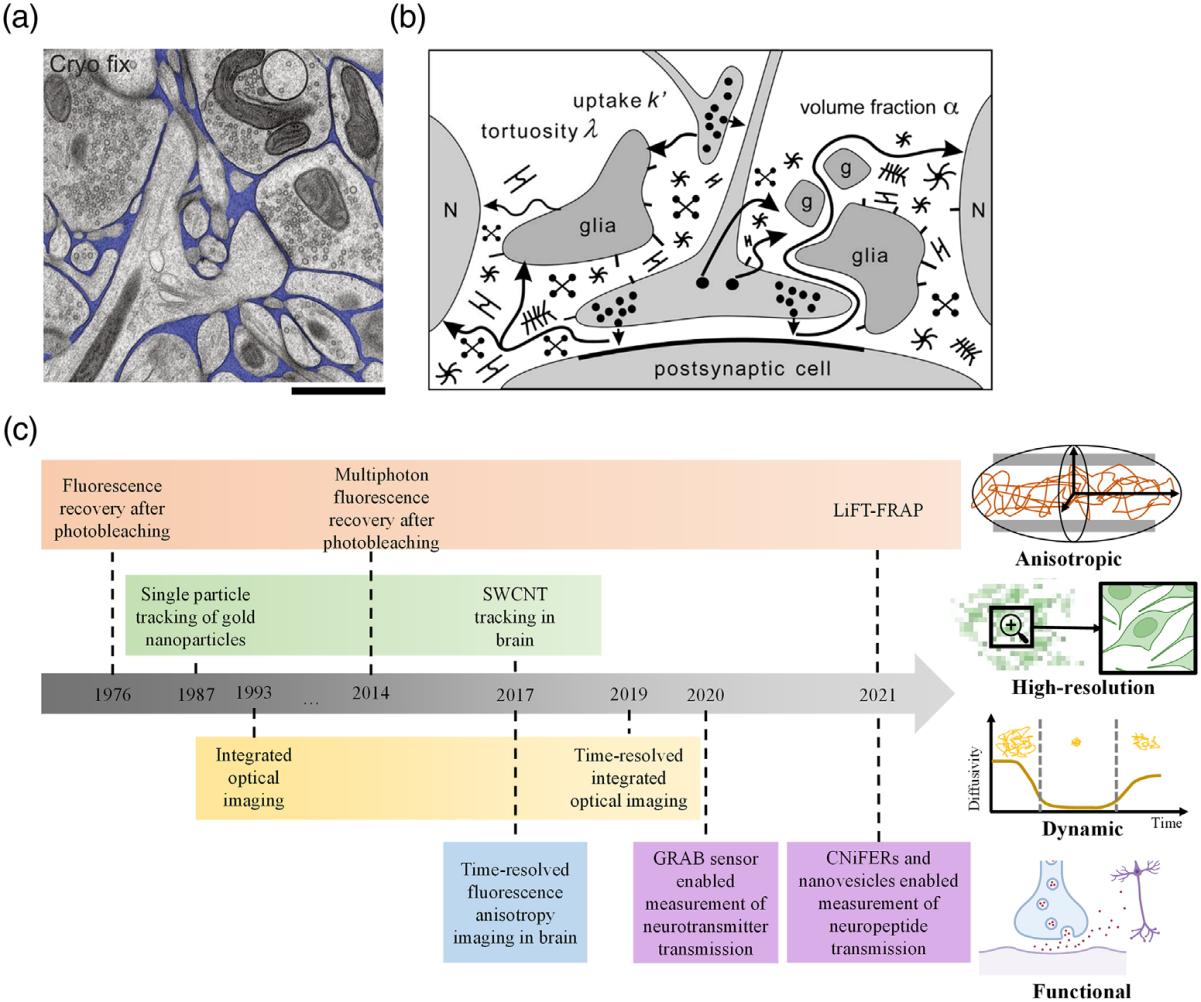
Brain ECS and diffusion measurement. (a) Electron microscopy image of cryofixed mouse cortex (reproduced with permission from Ref. 1). The ECS is colored in blue. The image clearly shows the brain ECS structure with the properties of the highly heterogeneous diameter and complex connectivity. Scale bar: 1  μm.^1^ (b) Schematic of substances diffusion in the ECS (reproduced with permission from Ref. 2). Multiple types of cells, ECM, and the geometry of interstitial channels can influence the diffusion process. The parameters that need to be considered when measuring the diffusion include tortuosity, cellular uptake, ECS volume fraction, etc.^2^ (c) Timeline of optical imaging techniques for diffusion measurement in brain ECS. Orange for FRAP, yellow for IOI, green for single particle tracking, blue for TR-FAIM, and purple for genetically modified methods. References for each technique: FRAP,^3^^,^^4^ single particle tracking of gold nanoparticles,^5^ IOI,^6^ multiphoton fluorescence recovery after photobleaching,^7^ SWCNT tracking in brain,^20^ TR-FAIM in brain,^8^ TR-IOI,^9^ GRAB sensor enabled measurement of neurotransmitter transmission,^10^ LiFT-FRAP,^11^ and CNiFERs and nanovesicles enabled measurement of neuropeptide transmission.^12^

Brain extracellular space (ECS) is the interstitial space between cell membranes, and it contains a solution that closely resembles the cerebrospinal fluid and extracellular matrix (ECM) molecules [Fig. 1(a)].^13^^,^^14^ The ECS occupies about 20% volume of the brain tissue, providing a reservoir with a number of substances that maintain brain cells activities.^15^ These substances include ions, such as $K^{+}$ and ${\mathrm{Ca}}^{2+}$, to maintain neuronal electrical activity;^16^ neurotransmitters for neuron-to-neuron communication; and neuromodulators, which use the ECS as the conduit for signal transmissions to other cells.^15^^,^^17^^,^^18^ The diffusion of substances in the ECS is limited by the geometry of the ECS [Fig. 1(b)],^2^^,^^15^^,^^17^^,^^19^^,^^20^ as well as the ECM, which consists of a dense mesh of glycoproteins, proteoglycans, and polysaccharide hyaluronan.^21^^,^^22^ Investigating molecular diffusion in the brain provides valuable information that can be used to help map the ECS structure, improve understanding of signaling processes and neurotransmitter function, and refine drug delivery to the brain.^23^^–^^25^

However, understanding the molecular transport in the brain ECS is challenging due to the complexity and dynamics of the ECS.^24^^,^^26^ To date, only a few methods have been developed to measure the extracellular diffusion, including diffusion weighted-magnetic resonance imaging (MRI), electrochemical method, and optical imaging. Although MRI is noninvasive and reaches a spatial resolution of up to 100  μm in the whole brain, it is limited to measuring the diffusion of water or contrast agents.^24^^,^^27^ The electrochemical method, for example, the real-time iontophoresis (RTI) coupled with ion-selective electrode detection, mainly measures the diffusion of tetra-methyl-ammonium (${\mathrm{TMA}}^{+}$), which is electroactive but physiologically inert.^28^^,^^29^ Although RTI provides quantitative measurement of the ECS diffusion, it has low spatial resolution and does not allow for visualizing the ECS. As an alternative, the optical imaging method utilizes fluorescent substances that diffuse in the ECS, allowing for real-time monitoring of their trajectories or fluorescent pattern by an advanced microscope [Fig. 1(c)]. For example, a single-walled carbon nanotube (SWCNT) with near-infrared (NIR) emission has been utilized as the fluorescent probe that can travel in the ECS for tens of minutes; a single SWCNT trajectory was recorded by a microscope with the localization approach, revealing the super-resolution structure of the ECS with 40 nm resolution.^20^ In addition to fluorescent substances, the study of endogenous nonfluorescent substances and their transmission through the ECS is important but has been limited by the lack of tools. This paradigm has started to change due to the availability of fluorescent sensors that specifically respond to different neurotransmitters and neuropeptides.^10^^,^^12^

In this review, we present an overview of recent advances in the optical imaging-enabled diffusion measurement in the brain ECS over last 5 to 10 years. Several comprehensive reviews by Nicholson and colleagues have provided a systemic overview of the ECS diffusion measurement techniques and properties.^13^^–^^15^ Here, we focus on recent optical imaging methods that allow for measurement of the dynamic, anisotropic, high resolution, and functional aspects of the brain ECS (Table 1). These include the use of advanced data analysis and imaging techniques, such as light-sheet and super-resolution imaging, and probes including fluorescent nanoparticles (NPs) and biosensors. We summarize the advantages and limitations of each method and give our perspective on future work in studying substances diffusion and signal transmission in the ECS.

**Table 1:** Summary of recent optical imaging enabled diffusion measurements.

Material		Molecular weight or particle size	Technique	Work condition	Spatial resolution	Temporal resolution	Application	Ref.
Fluorescent molecules	dextran	3 kDa	TR-IOI	Brain slice	NA	∼1 s	Dynamic diffusion coefficient during SD	9
	SF, FITC-dextran (FD)	SF: 376 Da, FD: 4 kDa, 10 kDa, 20 kDa,	LiFT-FRAP	Cornea *in situ*	NA	8 volumes/s, 64 images per volume	Noninvasive 3D anisotropic diffusion measurement	11
	Alexa Fluor 350	410 Da	TR-FAIM	Brain slice	NA	Line scanning rate up to 500 Hz	Nanoscale diffusion in synaptic cleft and in in a hippocampal slice	8
Fluorescent particles	SWCNT	5 nm diameter, 500 nm length	Particle tracking	Brain slice	∼40 nm	40 to 50 frames/s	Measuring the nanoscale dimensions and local viscosity of the ECS	20 and 30
	GPI–GFP labeled QD	30 to 35 nm diameter	Particle tracking	Brain slice	∼50 nm	30 frames/s	Measuring molecular dynamics of lipids and transmembrane proteins of synapses	31
	QD-WGA	39 nm diameter	Particle tracking	Brain slice	∼50 nm	20 frames/s	Revealing WGA transport in live brains	32
	PS−PEG	51 nm diameter	Particle tracking	Brain slice	NA	33 frames/s	Particle tracking with machine learning predicts neurodevelopmental age	33
Nonfluorescent substances	Acetylcholine, monoamines	<200 Da	Genetically encoded GRAB sensors	Brain slice	∼120 nm/pixel	10 to 50 Hz	Revealing the spread length constant of acetylcholine and monoamines	10
	SST	1639 Da	Nanovesicle release and SST2 CNiFER	Mouse cortex *in vivo*	1 μm/pixel	∼2 frames/s	Revealing the spatiotemporal scale of SST volume transmission	12

## 2 Diffusion Measurement of Fluorescent Molecules

### 2.1 Time-Resolved Integrated Optical Imaging

**Fig. 2:**
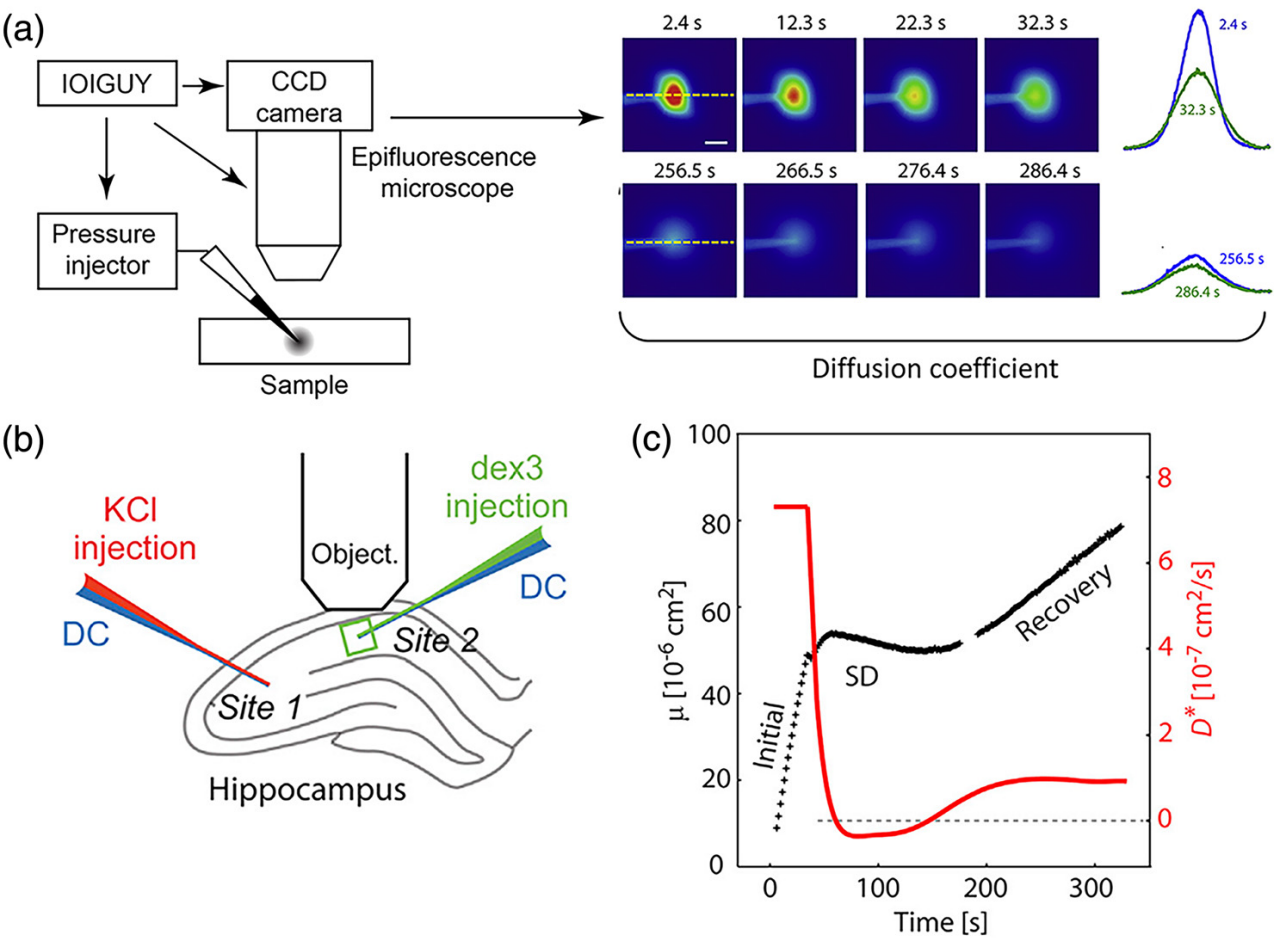
Optical measurement of fluorescent molecules diffusion by the IOI method. (a) Schematic of the IOI technique (adapted from Refs. 9 and 12). A small volume of probing molecule solution is injected into the sample. Then, a series of images are taken by a CCD camera attached to an epifluorescence microscope, capturing the diffusion cloud development of fluorescent molecules. Then by fitting the intensity profile along an axis from the recorded images to the diffusion equation, the diffusion coefficient is estimated.^9^^,^^12^ (b) Schematic of diffusion measurement in the SD induced hippocampus slice using the IOI system. Two double-barrel micropipettes are inserted: KCl is injected for SD induction; 3 kDa dextran is diffused from another site. Both sites are monitoring the SD progress by recording the DC potential.^9^ (c) The quantified profile of diffusion progress during the induced SD. Three phases during the SD progression observed by the time-resolved $D^{*}$ curve. Initial stage: $D^{*}$ holds stable; SD stage: $D^{*}$ decreased quickly to around zero, meaning the molecules stopped diffusion; recovery stage: molecules start moving with much smaller diffusion coefficient compared with the initial stage. The $D^{*}(t)$ is determined by derivative of μ(t). The dash line marks the $D^{*}=0$. Panels (b) and (c) reproduced with permission from Ref. 9.

The integrated optical imaging (IOI) system was first introduced by Nicholson and Tao^6^ and has been used to measure the diffusion of fluorescent molecules in the ECS for nearly 30 years. Molecules labeled with fluorophores are pressure injected into the brain tissue by a micropipette that is several micrometers in diameter, providing a point diffusion source. Then, time-lapse images are taken by an imaging system to record the fluorophores’ diffusion process [Fig. 2(a)], which is analyzed with a diffusion equation based on a model of diffusion from a point source [Eq. (1)]:^12^^,^^34^

$$C(r,t)=\frac{Q}{\alpha }\cdot \frac{1}{{\left(4D^{*}\pi t\right)}^{1.5}}\;\exp(-\frac{r^{2}}{2D^{*}t}-k^{\prime }t),$$ (1)

where the concentration C is a function of distance r and time t, Q is the total molecule number from the source, α is the volume fraction of brain ECS, $D^{*}$ is the effective diffusion coefficient, and $k^{\prime }$ represents the loss of signal caused by cellular binding, uptake, and clearance. As described in Fig. 2(a), to obtain the $D^{*}$, intensity profiles along one axis of the time-lapse images need to be extracted and fitted to the diffusion equation. Then, the diffusion permeability θ is written as $\theta =D^{*}/D$, where D is the free diffusion coefficient; the tortuosity λ is written as $\lambda ={\left(D/D^{*}\right)}^{1/2}$. Both θ and λ are used to characterize the impedance the tested molecule experienced in the obstructive medium.

Due to the complex environment and highly heterogeneous property of the ECS, all parameters are usually illustrated as volume averaged. One assumption is isotropic diffusion that assumes a homogeneous diffusion behavior in all directions from the fluorescent source point. Another assumption is that $D^{*}$ is a constant over time. Recently, Hrabe and Hrabetova imaged the diffusion of fluorescent dextran in rat brain slices during spreading depression (SD) with an advanced IOI system [Fig. 2(b)], improving temporal resolution from 10 s to ∼1  s.^9^ KCl was injected in the acute brain slice to induce the SD, and then 3 kDa fluorescent dextran was injected in the region of interest for diffusion recording. In this case, they considered the molecule concentration to be a function of position in space (r) and time (t) for presenting the time-resolved $D^{*}$. The time-dependent $D^{*}$ clearly reflects a three-phase change of the molecules’ diffusion in the ECS during the SD [Fig. 2(c)]. With above improvements on temporal resolution and the analysis method, such time-resolved IOI (TR-IOI) was able to capture rapid changes in the ECS diffusion within a dynamic environment. In a latest study, the group investigated diffusion permeability of 3 kDa dextran in the African naked mole-rat using the IOI method. They found that the ECS of the naked mole rats expands and preserves the molecular diffusion permeabilities under ischemia rather than shrinking for other rats.^35^

### 2.2 3D Fluorescence Recovery after Photobleaching with Light-Sheet Volumetric Imaging

Fluorescence recovery after photobleaching (FRAP) is a useful approach for the diffusion measurement of fluorescently tagged molecules in the micrometer scale regions of live specimens. A laser pulse is used to induce an irreversible photobleaching in the selected area, which is initially filled by fluorescent molecules. Then, fluorescence recovery in this selected area occurs over time as unphotobleached molecules from the surrounding area diffuse into the selected area.^36^ This results in a four-stage curve composed of the initial fluorescent signal, the photobleaching event, the recovery, and a final steady-state stage, respectively. Compared with the initial intensity, the final stage intensity may not recover 100% of the initial intensity because some fluorescent molecules in the surrounding area are immobile. The intensity curve of the recovery and final stage is fitted with an exponential function to yield a half time ($\tau_{1/2}$), which relates to the diffusion coefficient of the substance. Although previous efforts have been made to use FRAP to measure the macromolecular diffusion by cortical surface photobleaching^37^^,^^38^ or in deep tissue with optical fibers,^39^ the robustness and ability to provide quantitative diffusion coefficients have been questioned.^15^ Although FRAP has some potential for the ECS diffusion measurement, there are several challenges. First, FRAP has found success in measuring fluorescent molecule diffusion in the two-dimensional (2D) lipid membrane, whereas measuring diffusion in three-dimensional (3D) requires a well-defined and large photobleaching volume. Furthermore, it requires techniques that can image the fluorescent recovery progress with deep tissue penetration and ideally with 3D volume imaging.

**Fig. 3:**
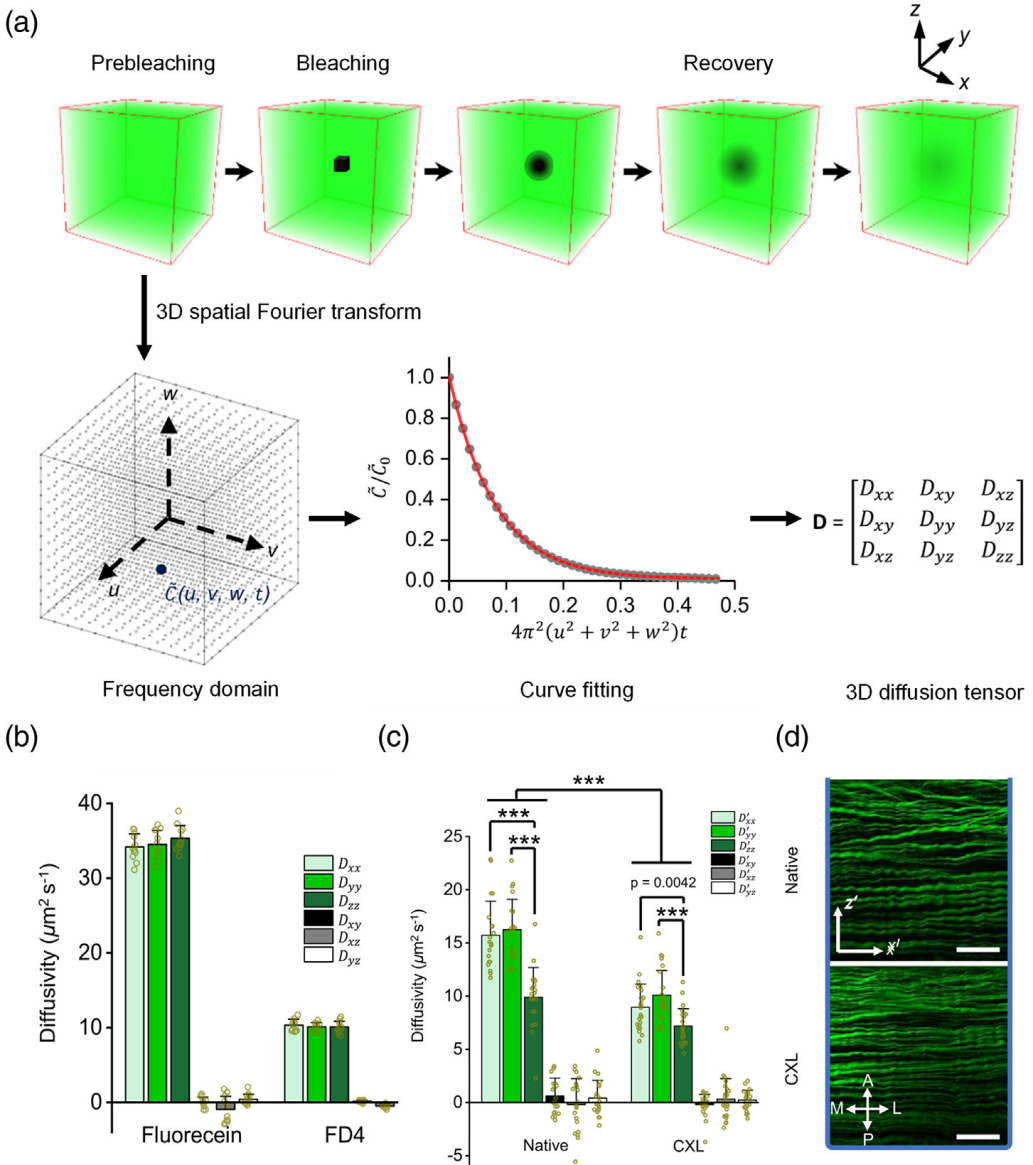
Optical measurement of fluorescent molecules diffusion by the LiFT-FRAP method. (a) Schematic of the LiFT-FRAP experiment workflow. Time series 3D images are recorded and converted to the frequency domain by 3D spatial Fourier transformation. The normalized fluorophore concentration $\tilde{C}/{\tilde{C}}_{0}$ decreases with the fluorescence recovery in the frequency domain (u, v, and w are the spatial frequency coordinates). The data are fitted to obtain the diffusion coefficient D and the 3D diffusion tensor. (b) Directional diffusivity in 3D diffusion tensor of sodium fluorescein and 4 kDa fluorescein isothiocyanate-conjugated dextran in free diffusion medium. (c) 3D diffusion tensor components of 20 kDa fluorescein isothiocyanate-conjugated dextran in native and collagen crosslinking treated corneas. (d) 2D second-harmonic generation imaging of native and collagen crosslinking treated corneas. The x and y directions are parallel to the laminar structure; the z direction is perpendicular to the laminar structure. Scale bar: 50  μm. A, anterior; P, posterior; M, medial; L, lateral.^11^ (a)–(d) Reproduced with permission from Ref. 11.

Recently, Chen *et al*. developed a noninvasive technique named light-sheet imaging-based Fourier transform fluorescence recovery after photobleaching (LiFT-FRAP) that seems to address these challenges. They used a two-photon 3D volume bleaching generator to create a confined 3D bleaching with high speed two-photon scanning light-sheet microscopy. This system allows for fast imaging of the 3D volume to measure the 3D diffusivity of the cornea and quantify the anisotropy of the diffusion in different directions.^7^^,^^11^ The acquired time-series images are processed by 3D spatial Fourier transform and then a normalized concentration profile of fluorophores is obtained in the frequency domain [Fig. 3(a)]. To acquire anisotropic diffusivity, they considered the diffusivity to be a 3D tensor. The diagonal elements of the tensor ($D_{xx}$, $D_{yy}$, $D_{zz}$) indicate the diffusivity in three dimensions along the Cartesian coordinate axes. Diffusion is anisotropic when having different diffusivities for the three directions. Figure 3(b) shows the directional diffusivities of sodium fluorescein and fluorescein-dextran in a 60% glycerol solution. The directional diffusivities are similar in all three directions, suggesting isotropic diffusion. In contrast, fluorescein-dextran diffuses faster in the direction that is parallel to the lamellar structure of the cornea (x and y directions) than the perpendicular direction (z direction) [Figs. 3(c) and 3(d)]. Impressively, this technique integrated FRAP and light-sheet microscopy, combining their respective advantages of a large bleach volume ($1000\;\mu m^{3}$) and fast 3D imaging. This platform provides a noninvasive method to measure the 3D diffusion tensor and can be useful for measurement of different tissue types such as the brain and spinal cord, where the tissue is highly heterogeneous.

### 2.3 Time-Resolved Fluorescence Anisotropy Imaging

**Fig. 4:**
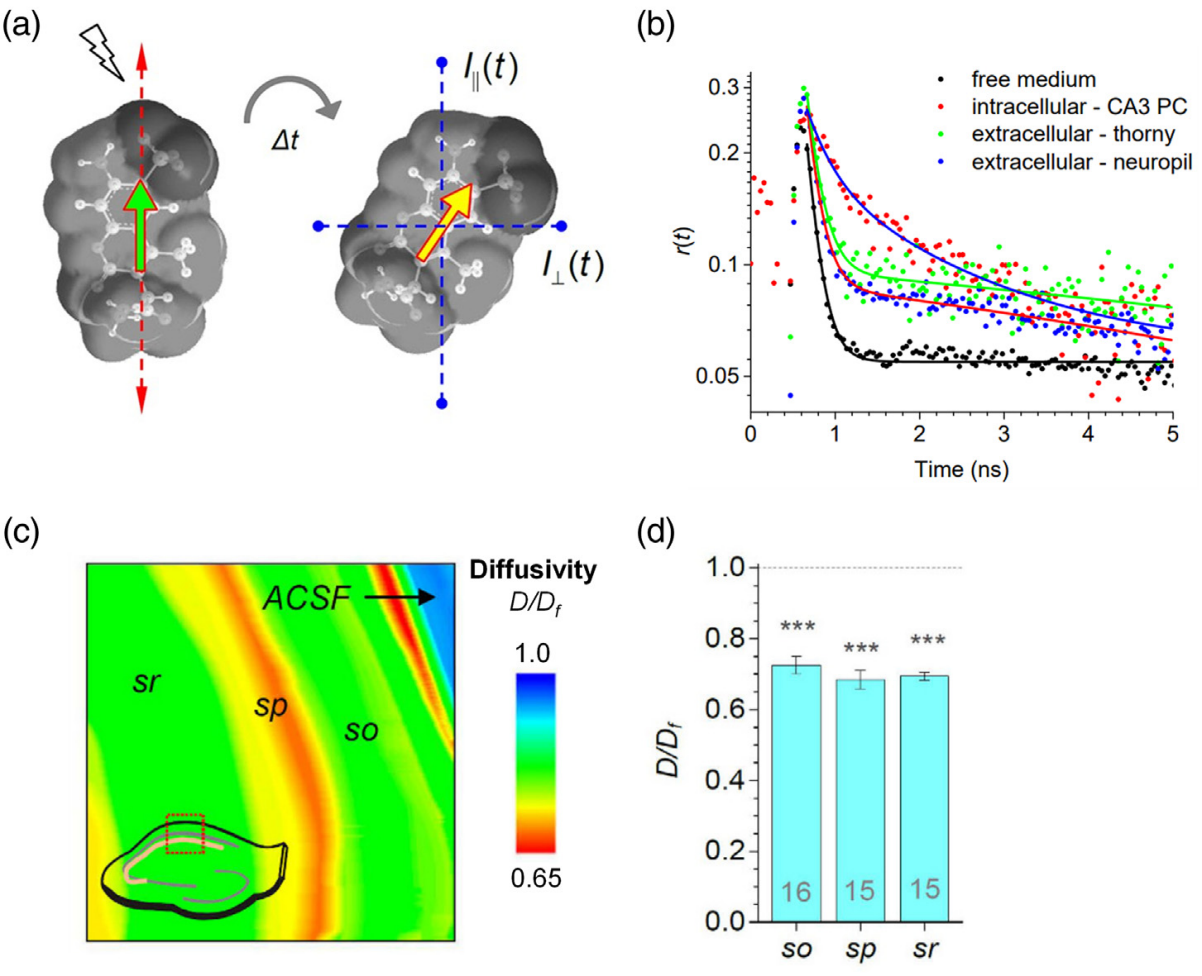
Optical measurement of fluorescent molecules diffusion by the TR-FAIM method. (a) Theory of Alexa Fluor 350 anisotropy imaging. The fluorophore excitation plane (green arrow) aligns to emission polarization plane (red arrows), while the deviation caused by molecule motion over time Δt, and the emission is recorded by detectors $I_{∥}(t)$ and $I_{⊥}(t)$. (b) Example of fitted anisotropy decay curves of Alexa Fluor 350 diffusion in free medium, intracellular space, and ECS. (c) Example map of Alexa Fluor 350 $D/D_{f}$ in the CA1 region of the acute hippocampus slice. (sr, stratum radiatum; sp, stratum pyramidale; so, stratum oriens; ACSF, free medium). (d) Summary of averaged $D/D_{f}$ values corresponding to the different subregions in (c).^8^ (a)–(d) Reproduced with permission from Ref. 8.

Fluorescence anisotropy imaging is a technique that is usually applied to live cells, contributing especially to fluorophores viscosity and diffusion rate investigation.^40^^,^^41^ Time-resolved fluorescence anisotropy imaging (TR-FAIM) was reported by Zheng et al. for 2D intercellular diffusivity mapping in acute brain slices.^8^^,^^42^ This technique was combined with two-photon microscopy to map extracellular nanoscale diffusivity of acute hippocampal slices. During the time interval between fluorophore excitation and emission, the fluorophore’s motion would cause the deviation of the emission polarization plane from the excitation polarization plane. The fluorescence decay was recorded by two perpendicular polarization detectors [Fig. 4(a)]. Then, the fluorescence anisotropy time course r(t) was obtained and fitted [Fig. 4(b)]. Zheng et al. measured the diffusion of Alexa Fluor 350 in rat brain slices and found two types of diffusion behaviors. The fast one with a decay time between 0.2 and 0.4 ns corresponds to the interstitial free diffusion; the slow one with a decay time between 2 and 12 ns indicates the restricted movement due to cellular binding and other immobilization. The effective-to-free diffusion coefficient ratio ($D/D_{f}$) is analyzed for each pixel. Figure 4(c) shows the diffusivity ratio map through the CA1 region of a hippocampal slice. The result suggests that the Alexa Fluor 350 diffusion in the hippocampus is around 30% slower than that in the free medium overall, with a small deviation among the subregions [Fig. 4(d)]. This method provides a good description of molecule diffusion through a piece of tissue, shows the subregional differences in diffusion, and can identify restricted motion within the brain ECS.

## 3 Diffusion Measurement of Fluorescent Nanoparticles

Limited by the light diffraction-limit, the spatial resolution of conventional fluorescent microcopy used in IOI or FRAP is on the order of 0.5  μm. In contrast, the spatial resolution of single particle tracking technique can achieve as high as tens of nanometers, which can provide more information not available by IOI or FRAP. Single particle tracking was first tried on colloidal gold nanoparticles with a diameter of 40 nm by video-enhanced contrast light microscopy.^5^ Since then, the technique has been used for various *in vitro* and *in vivo* applications,^43^ including the diffusion measurement in the brain ECS. Fluorescent nanoparticles (NPs) are employed to measure the diffusion in the brain ECS due to their high brightness and fluorescent stability. The measurement is usually based on single-particle tracking, which is similar to the localization-based super-resolution approach. The Brownian motion of a single fluorescent NP in the ECS is recorded by an imaging system and thousands of images encoding NP positional localization are generated during tens of minutes of acquisition time. These images are first drift corrected to eliminate microscope drift error with the aid of a cross-correlation algorithm.^44^ Afterward, the position of the NP in each image is close to the center of the point spread function from Gaussian fitting, affording significantly enhanced resolution.^45^ After applying this process to all images, the positional centers are used to reconstruct an image with high resolution. Moreover, since the positional center of the NP is known at each time point its trajectory and diffusion coefficient are plotted and analyzed. Note that the single-particle tracking technique requires an extremely bright and photostable fluorescent NP to maximize the signal-to-noise ratio of the image and decrease photobleaching during the long acquisition time.

### 3.1 Single-Walled Carbon Nanotube

The SWCNT has two advantages in single-particle tracking measurements in the brain ECS. First, the dimensions of an SWCNT are typically several nanometers in diameter and 5 to 10  μm in length, resulting in a moderate diffusion rate in brain ECS that can be facilely captured by an imaging system.^46^ Second, once excited with NIR light, the fluorescent emission of an SWCNT is also in the NIR window. This can significantly improve imaging depth up to 1 mm in the brain tissue.^47^^,^^48^

**Fig. 5:**
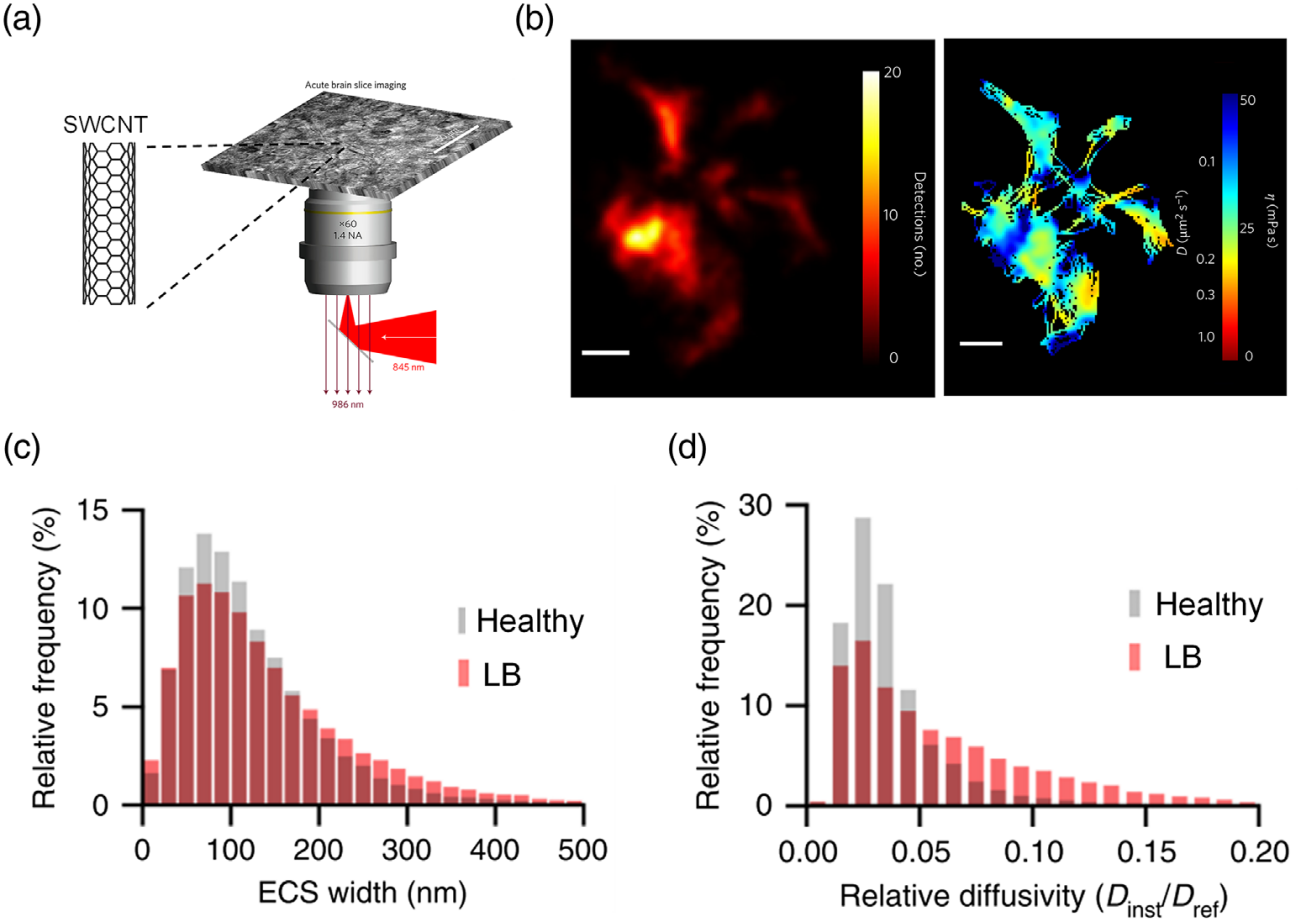
Optical measurement of SWCNT diffusion. (a) SWCNTs are imaged using a wide-field fluorescent NIR microscope in acute brain slices. The wavelength of excitation is 845 nm, and emission is at 986 nm (adapted from Ref. 20). (b) Left: super-resolution imaging of live brain ECS morphology map. Color bar represents the frequency of the detected nanotube. Right: map of the instantaneous diffusion coefficients and local ECS viscosity (reproduced with permission from Ref. 20). Scale bar: 500 nm.^20^ (c) Frequency distribution of width and (d) local relative diffusivity of the ECS of SN in healthy and LB-inoculated mice (reproduced with permission from Ref. 30).

Godin et al. utilized a phospholipid–polyethylene glycol (PL–PEG) coated SWCNT as the tracking agent to discover the ultrastructure of the brain ECS.^20^^,^^49^ They injected SWCNTs into the rat lateral ventricles, and the rat brain was sliced into acute brain slices with 0.5 mm thickness [Fig. 5(a)]. Then, the acute brain slice was imaged with an NIR microscope to collect over 20,000 frames, which were used to reconstruct a super-resolution image and analyze diffusion properties of the SWCNT [Fig. 5(b), left]. Impressively, the super-resolution images indicated that the brain ECS is highly heterogeneous with a broad distribution of widths varying from ∼50 to 700 nm. The SWCNT’s viscosity map confirmed that diffusion in the brain ECS is highly heterogeneous [Fig. 5(b), right]. Moreover, the dimension of the ECS and the diffusion properties of SWCNT in the ECS may be dramatically altered in brain diseases. Soria et al. used this single SWCNT tracking technique to interpret how the ECS changes in Parkinson’s disease (PD) mice.^30^ They induced the PD in mice by inoculating the Lewy body (LB) fractions derived from PD patients into the substantia nigra (SN) of adult mice. They found that under PD pathological context, the ECS width distribution in SN appears larger than the width distribution obtained from healthy mice [Fig. 5(c)]. The instantaneous relative diffusivity ($D_{\mathrm{inst}}/D_{\mathrm{ref}}$) of the SWCNT, where $D_{\mathrm{ref}}$ is the free medium diffusivity, increased with respect to that of SWCNTs in healthy mice [Fig. 5(d)]. These findings can be ascribed to the fact that the hyaluronan in the ECS is depleted during the development of PD.

The single SWCNT tracking technique can resolve the super-resolution structure of the brain ECS, but it has some limitations. The image acquisition is time-consuming. An acquisition time for single SWCNT tracking measurement is about 10 to 20 min, and hundreds of acquisitions are required for one data set. In addition, the large size (diameter: 5 nm; length: 500 nm, Table 1) could lead to many SWCNTs injected into the brain being stuck in the ECS, complicating the experiment. More work is necessary to simplify and disseminate these techniques.

### 3.2 Quantum Dot

**Fig. 6:**
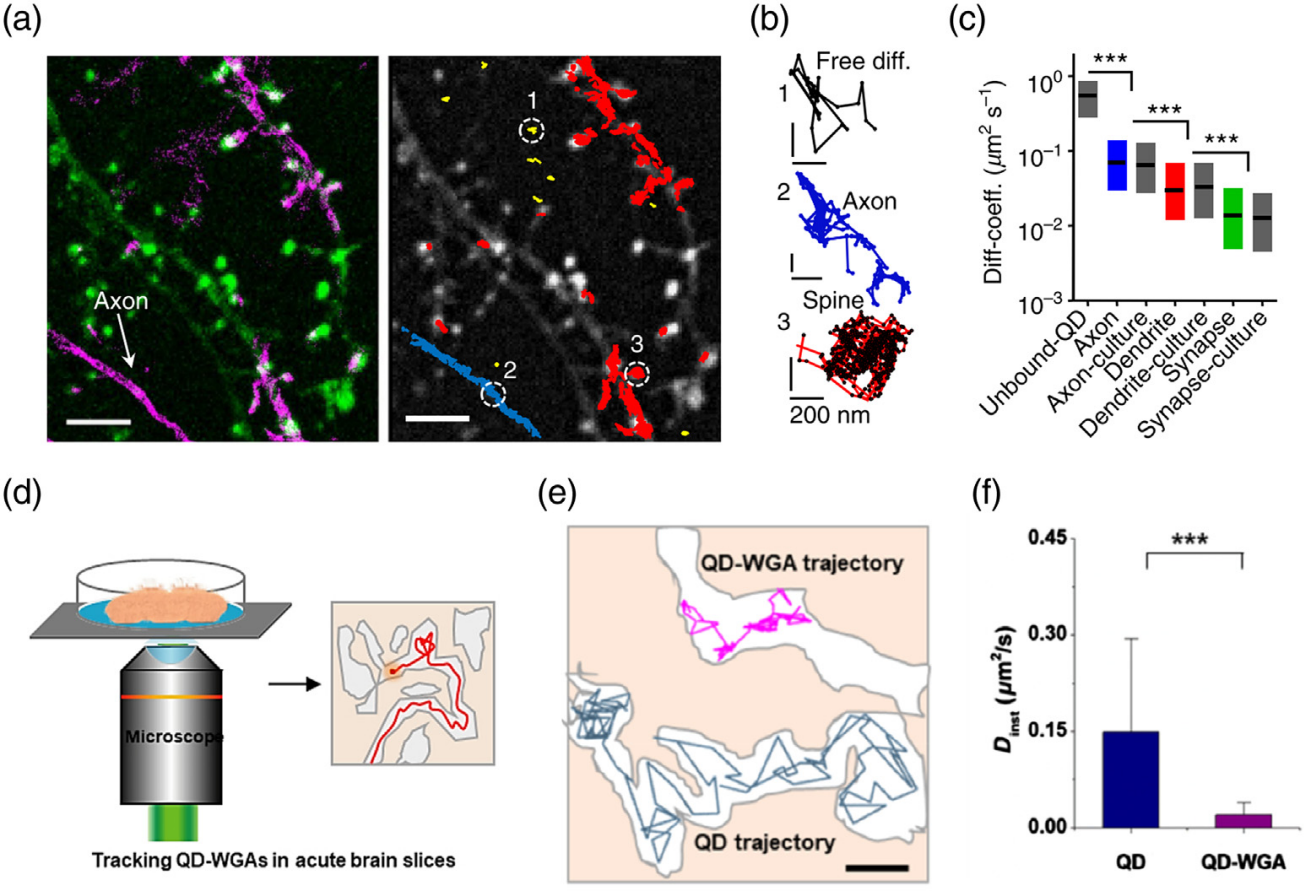
Optical measurement of QDs diffusion. (a) Left: image of dendrites (green) and GPI-GFP (magenta). Right: trajectories of individual QD-labeled GPI-GFP dynamics within different structures (red, dendritic; blue, axonal; yellow, extracellular). Scale bar: 10  μm. (b) Individual trajectories of QD-labeled GPI-GFP from marked areas in panel (a): extracellular free diffusion (black), axonal (blue), and spine (red) surface dynamics; scale bar: 200 nm. (c) Diffusion coefficient of GPI-GFP within ECS and multiple subcellular compartments.^31^ (a)–(c) Reproduced with permission from Ref. 31. (d) Schematic of real-time individual QD-WGAs tracking in acute brain slices. (e) Example diffusion trajectories of single QD-WGA (magenta) and QD (dark blue) in brain ECS. Scale bar: 2  μm. (f) The instantaneous diffusion coefficients of QD and WGA-QD in the ECS. (QD, $D_{\mathrm{inst}}=0.15\pm 0.15\;\mu m^{2}/s$; QD-WGA, $D_{\mathrm{inst}}=0.021\pm 0.019\;\mu m^{2}/s$).^32^ (d)–(f) Reproduced with permission from Ref. 32.

Quantum dot (QD) has been frequently used in single-particle tracking because it has a high quantum yield (usually >0.8) and small size. Biermann et al.^31^ used antibody coupled QDs to target the glycosylphosphatidylinositol (GPI) on neurons and glia in the brain slice and monitor the dynamics of GPI by recording the motion of QDs. The antibody-coupled QDs with a size of 30 to 35 nm were incubated with the brain slice instead of injecting them into the brain because the smaller size of QDs allows them to penetrate the brain and diffuse in the ECS. Figures 6(a)–6(c) show that the trajectories and diffusivities of QDs along axons, dendrites, and synapses exhibited different dynamics. More recently, Wang et al. used QD-labeled wheat germ agglutinin (QD-WGA) to analyze the mobility behavior of WGA in the ECS of brain slices after intracerebroventricular injection [Fig. 6(d)].^32^ WGA is an effective antitumor drug and axonal transport carrier that facilitates drug delivery into the brain. Figures 6(e) and 6(f) show that the instantaneous diffusion coefficient of QD-WGA in the brain ECS is sevenfold lower than that of free QDs, suggesting that WGA molecules have a strong interaction with brain cells. With the formation of fluorescent self-interference (SELFI), Linarès-Loyez et al.^50^ developed a method to track a single QD in 3D at a high frame rate (e.g., 50 frames per second), which offers a powerful tool to study the molecular nano-organization and dynamics in complex samples in which 2D imaging-only can lead to biased results. One potential problem is that the inherent photoblinking of QDs may cause false localization in single-particle tracking experiments.^51^

### 3.3 Polymeric Nanoparticles

**Fig. 7:**
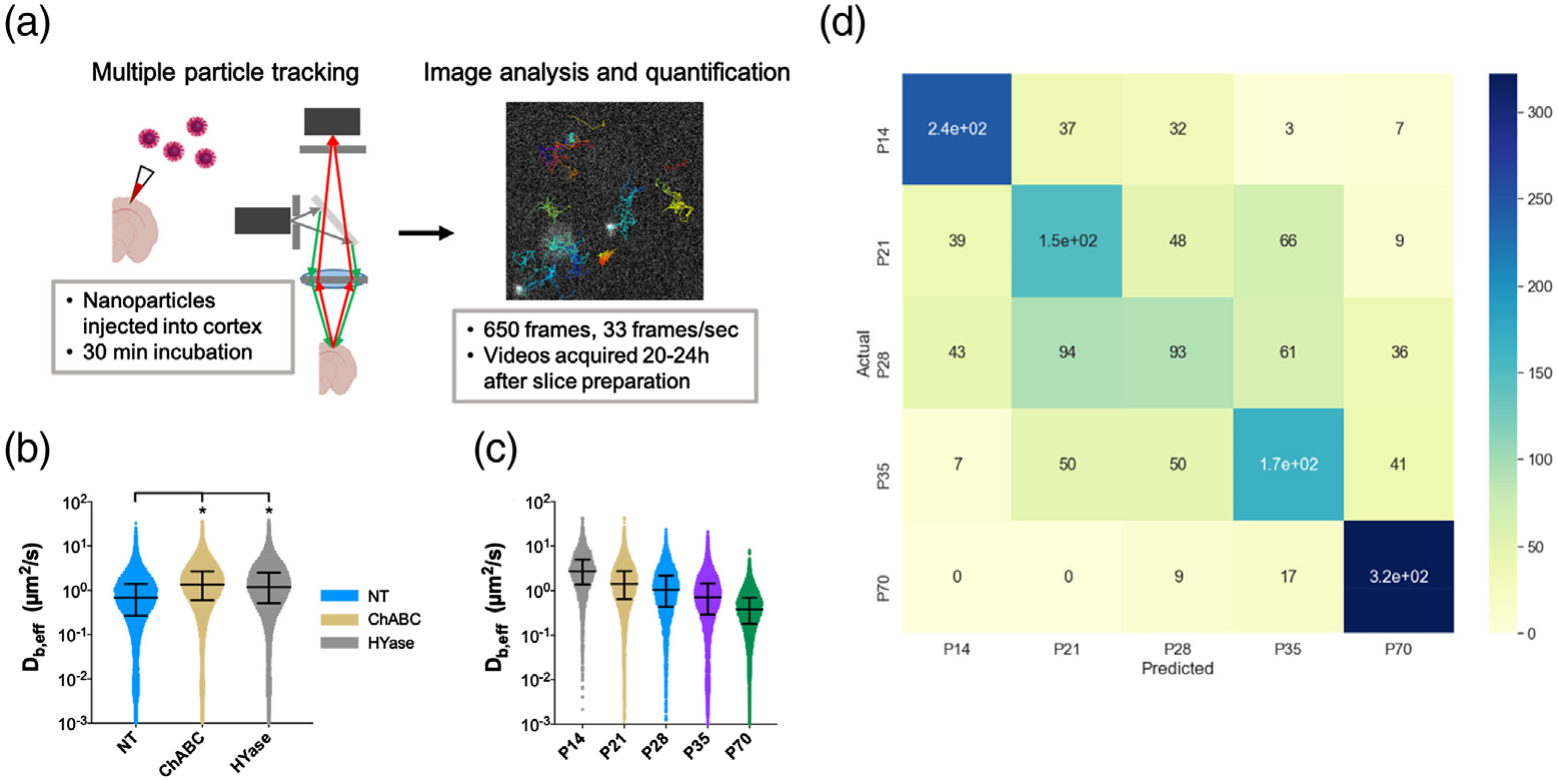
Optical measurement of PS-PEG diffusion. (a) Workflow of multiple particle tracking experiment and data analysis. PS–PEG (40 nm) NPs were injected into the 300-μm-thick organotypic brain slices; then images were collected after 30 min incubation. (b) Distribution of $D_{b,\mathrm{eff}}$ (diffusivity in cortical ECM) values for each postnatal age. (c) Distribution of $D_{b,\mathrm{eff}}$ values in nontreated (NT) (blue), ChABC-treated (yellow), and HYase-treated (gray) P35 brain slices. (d) Confusion matrix of predicted age versus actual age for test data sets. The color bar represents the number of trajectories.^33^ (a)–(d) Reproduced with permission from Ref. 33.

Polyethylene glycol (PEG) coating is a well-known strategy for significantly enhancing NP penetration and diffusion in the brain because neutral PEG coating avoids adhesive interaction with the surrounding environment.^52^ With 51-nm dense PEG-coated polystyrene (PS-PEG) NP, McKenna et al. observed the structural changes of brain ECM during neurodevelopment and predicted the neurodevelopmental age by a machine learning method [Fig. 7(a)].^33^ Furthermore, the hyaluronan scaffolds in the ECS were depleted by either ChABC or Hyase enzyme, resulting in the PS-PEG NP diffusing faster in the brain ECS [Fig. 7(b)]. They also conducted PS-PEG NP tracking experiments on mice of different ages, from P14 to P70, and showed that the diffusion coefficient decreased with age [Fig. 7(c)]. By collecting a large amount of data on different aged mice, they extracted 39 different features from trajectories and trained a machine learning model for age stages classification. As shown in Fig. 7(d), the model predicted the lower and upper limit age stages P14 and P70 with high accuracy and a recall of 74.47% and 84.40%, respectively. Although the accuracy in predicting the medium age stages (P21, P28, P35) surpassed that of random guessing (20%), the model was unable to classify these cases with high accuracy. This work suggests that the diffusion of NPs in the ECS could be used for chronological age verification.

## 4 Diffusion Measurement of Nonfluorescent Substances

The transport of biomolecules in the brain ECS is essential for intercellular communication and brain homeostasis.^13^^,^^14^ For instance, the transmission and distribution of excitatory, inhibitory neurotransmitters, and neuromodulators have a big impact on the functions of the neuronal network.^18^^,^^25^^,^^53^ However, the diffusion and distribution of these neurotransmitters and neuromodulators are limited by the visualization tools. Endogenous biomolecules or delivered drug substances are usually nonfluorescent. Moreover, the local concentration for some neurotransmitters is very low in the range of nM to μM, and the diffusion distance ranges from nanometer within synaptic cleft to micrometer or millimeter covering a number of neurons.^54^^,^^55^ These factors make it challenging to quantitatively determine the diffusion properties of these neurochemicals. To date, various fluorescent indicators have been developed for nonfluorescent substances detection. Such indicators are categorized by two scaffolds: G-protein coupled receptors (GPCR) or microbial periplasmic binding protein, as reviewed by Tian et al.^56^ Since most of current studies focus on the optical measurement of the spatiotemporal dynamics of neurochemical release, the mapping of their diffusion in the ECS is still at the early stage.

**Fig. 8:**
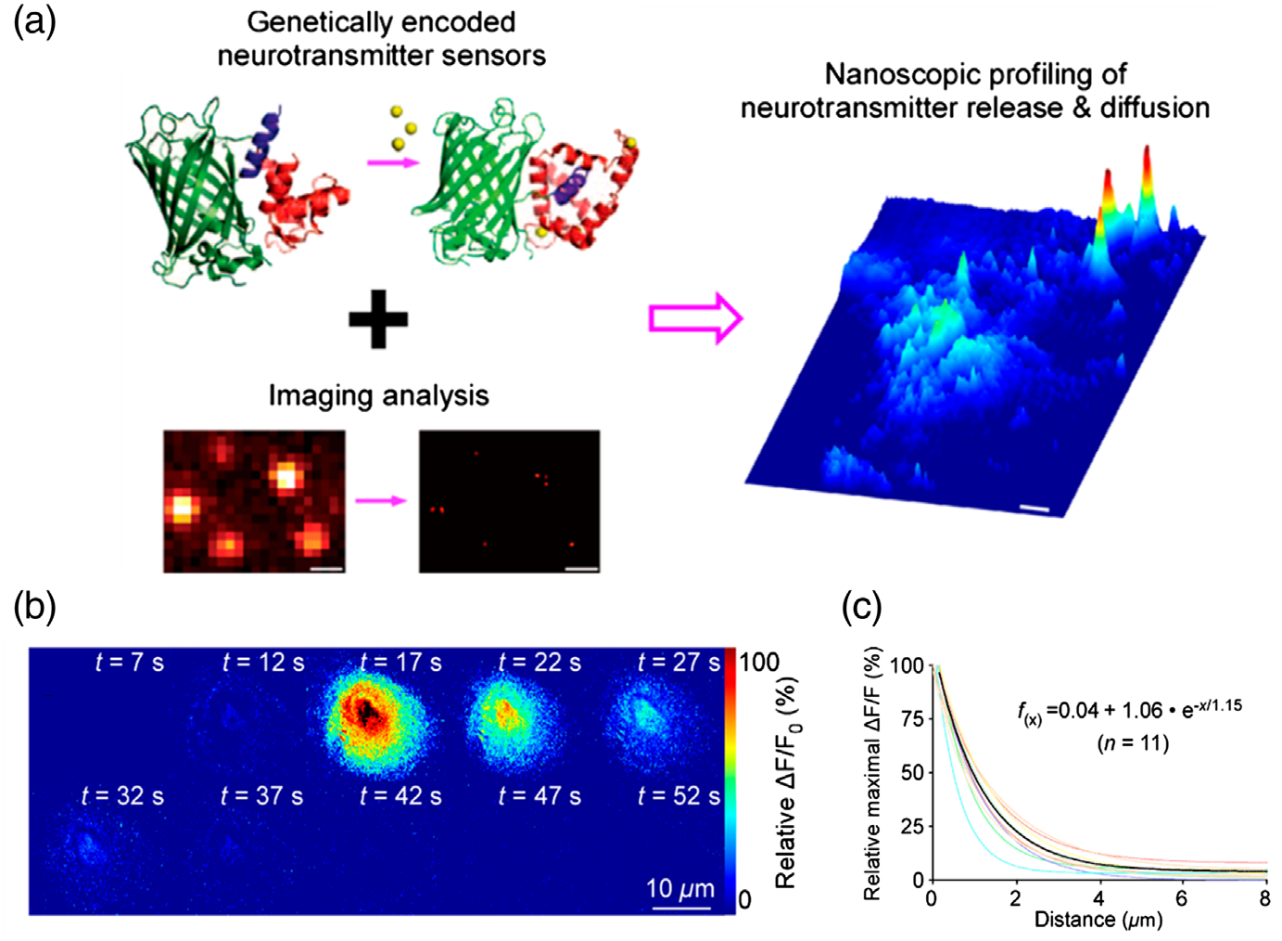
Neurotransmitter diffusion measurement by genetically encoded fluorescent sensors. (a) Visualization of neurotransmitter diffusion at various cells by combining genetically encoded fluorescent sensors with optical imaging and analysis algorithms. (b) Heatmap of time-series 2D $\Delta F/F_{0}$ response profiles adrenergic transmission at amygdalar neurons after electrical stimuli in GRABNE1m-expressed brain slice. Scale bar: 10  μm. (c) Plot of relative fluorescence decay versus distance from putative single release sites. The spread length constants are obtained at the distance of 50% decay. The fitting curve of decay function is in black.^10^ (a)–(c) Reproduced with permission from Ref. 10.

Yulong Li’s group and Lin Tian’s group have recently developed genetically encoded GPCR-based fluorescent sensors named GRAB and Light, respectively, for several neurotransmitters such as dopamine,^57^^–^^59^ norepinephrine (NE),^57^^,^^60^ and serotonin.^57^^,^^61^ The fluorescent protein EGFP is inserted into the intracellular loop of GPCRs, resulting in the fluorescence intensity increase activated by the receptor binding. With the GRAB sensors and high-resolution imaging, Zhu et al. visualized the local distribution of endogenously released neuromodulators diffusion into the ECS and analyzed their spread length constant at both neuronal and non-neuronal cell sites [Fig. 8(a)].^10^ For adrenergic transmission, ${\mathrm{GRAB}}_{\mathrm{NE}1m}$ has been used for NE detection. After the fluorescent sensor expression, the local electrical stimuli were applied to acute mouse amygdalar slices to induce endogenous NE release. They found that the spatial $\Delta F/F_{0}$ response is time-dependent [Fig. 8(b)]. They chose the site around the evoked neuron with the highest $\Delta F/F_{0}$ response as the neurotransmitter release source for diffusion analysis. The average NE spread length constant is ∼1.2  μm at amygdalar neurons [Fig. 8(c)]. Diffusion of multiple types of neurotransmitters has been measured with various genetically encoded sensors, including acetylcholine, serotonin, and dopamine. The spread length constants of these electrically evoked neurotransmitters’ responses vary in the range from 0.75 to 1.3  μm. This study proves that spatially restricted transmission is an important mode of cell-to-cell communication among various neuromodulators. Such microscopic visualization and characterization method provide a robust way to analyze the various neuromodulators transmission for different brain regions and cell types.

In contrast to the classical synaptic transmission, neuropeptides can diffuse from axons and signal through GPCRs at relatively long distances. This diffusion-driven distribution is referred to as volume transmission, an extrasynaptic dispersion of transmitter in the ECS.^18^ Determining where and when a neuropeptide acts relative to its release site provides a critical link to understanding its functional role in controlling neural circuits. Recently, Xiong et al. developed an optical approach to detect neuropeptide diffusion in the mouse neocortex. Somatostatin-14 (SST) was encapsulated in plasmonic nanovesicles (Au-nV-SST) and released by NIR laser pulses stimulation.^12^ The released SST was detected by a cell-based neurotransmitter fluorescent engineered reporter (SST2 CNiFER) with nM sensitivity. CNiFERs utilize a clonal HEK2893 cell that is engineered to express a specific GPCR and fluorescence resonance energy transfer-based ${\mathrm{Ca}}^{2+}$ sensor. Taking advantage of plasmonic nanovesicles and optical neuropeptide sensor, the integrated approach reveals the spatiotemporal scale of neuropeptide transmission and signaling *in vivo*. This approach could have further applications with multiple brain regions diffusion characterization of healthy and diseased mouse.

## 5 Discussion and Perspective

Compared with other approaches, optical imaging is a straightforward approach for diffusion studies with high compatibility to various molecules size and types. New developments of conventional techniques, such as TR-IOI and LiFT-FRAP, have been able to capture the dynamic and 3D anisotropic diffusion properties of fluorescent molecules. Several fluorescent nanoparticles, such as SWCNT, QDs, and polymeric nanoparticles, have been used for optical particle tracking with high spatial and temporal resolution to investigate the nanoscale dimensions of brain ECS and their local diffusivity. The use of advanced data analysis, cutting-edge imaging techniques, and functional probes measured the dynamic, anisotropic, high resolution, and functional aspects of the brain ECS.

There are several areas for future studies. First, further work to correlate different measurement methods is required to reach consensus on ECS geometry. IOI measures the diffusion of QDs and predicted a characteristic ECS width of 38 to 64 nm.^62^ Particle tracking in acute brain slices shows that the ECS width varies from 50 to 400 nm, half of which are between 80 and 220 nm.^49^ Electron microscopy imaging of high-pressure cryofixed brain tissue shows that the width of the ECS is highly heterogeneous and can reach 400 nm, with 50% ECS widths smaller than 100 nm.^30^ The deviations in these results are potentially due to sample preparation differences and imaging resolutions. The ECS of organotypic slices used by some of these studies may be slightly different compared with *in vivo* conditions or acute brain slices.^49^ Current super-resolution optical imaging in brain tissue is limited to 50 nm, compared with much higher resolution in EM imaging. EM imaging with the high pressure cryofixation is the best choice currently for maintaining the cellular structure, so it could be considered to be the gold standard for the ECS width analysis. Future work on super-resolution imaging *in vivo* is of interest for examining the tissue under intact conditions, but it may be challenged by blood flow and respiration-induced motions.

Second, current measurements of the ECS diffusion focus on selected areas in the brain. With advances in high throughput imaging, it may be possible to consider mapping the ECS diffusion across the brain in healthy and disease conditions. This may lead to new insights into how the ECS geometry and ECM change in diseases such as brain tumor and how it impacts therapeutic drug delivery.

Third, in addition to investigating the structural properties of the brain ECS, an area of significant interest is to study the functional properties, especially the endogenous neurochemical transmission in the brain. The intercellular transmission and signaling through brain ECS are extremely important for the activity of the mammalian brain. However, understanding these processes is still a significant challenge in neuroscience. For example, how far and fast most neuropeptides can diffuse after release are barely understood and represent obstacles to elucidating their function role in healthy and diseased neural circuitry. The rapid development of genetically encoded fluorescent biosensors provides promising tools to detect neurochemicals with high sensitivity and specificity. The capability to sense the neurochemicals with high spatiotemporal resolution will advance our understanding of the intercellular communication by neurotransmitters and neuromodulators including neuropeptides. We envision more efforts to measure the neurochemical transmission in the brain due to availability of fluorescent sensors, a challenging but extremely important area due to the physiological signaling processes and their functions in the brain circuits. Toward this, photosensitive NPs including plasmonic nanovesicles show promise in the diffusion measurement. The fast release of specific neuropeptides from plasmonic nanovesicles can mimic the endogenous release from axons, making it possible to study the neuropeptide volume transmission. The functional NPs are expected to have more important applications in understanding brain ECS diffusion and signaling in the future.

## References

[1] Korogod N. Petersen C. C. Knott G. W., “Ultrastructural analysis of adult mouse neocortex comparing aldehyde perfusion with cryo fixation,” eLife 4, e05793 (2015). 10.7554/eLife.05793

[2] Sykova E., “Extrasynaptic volume transmission and diffusion parameters of the extracellular space,” Neuroscience 129(4), 861–876 (2004). 10.1016/j.neuroscience.2004.06.077 15561404

[3] Axelrod D. et al., “Mobility measurement by analysis of fluorescence photobleaching recovery kinetics,” Biophys. J. 16(9), 1055–1069 (1976). BIOJAU 0006-3495 10.1016/S0006-3495(76)85755-4 786399 PMC1334945

[4] Koppel D. et al., “Dynamics of fluorescence marker concentration as a probe of mobility,” Biophys. J. 16(11), 1315–1329 (1976). BIOJAU 0006-3495 10.1016/S0006-3495(76)85776-1 974223 PMC1334960

[5] Geerts H. et al., “Nanovid tracking: a new automatic method for the study of mobility in living cells based on colloidal gold and video microscopy,” Biophys. J. 52(5), 775–782 (1987). BIOJAU 0006-3495 10.1016/S0006-3495(87)83271-X 3427186 PMC1330181

[6] Nicholson C. Tao L., “Hindered diffusion of high molecular weight compounds in brain extracellular microenvironment measured with integrative optical imaging,” Biophys. J. 65(6), 2277–2290 (1993). BIOJAU 0006-3495 10.1016/S0006-3495(93)81324-9 7508761 PMC1225970

[7] Shi C. et al., “Measurement of three-dimensional anisotropic diffusion by multiphoton fluorescence recovery after photobleaching,” Ann. Biomed. Eng. 42(3), 555–565 (2014). ABMECF 0090-6964 10.1007/s10439-013-0939-7 24248560 PMC3943478

[8] Zheng K. et al., “Nanoscale diffusion in the synaptic cleft and beyond measured with time-resolved fluorescence anisotropy imaging,” Sci. Rep. 7, 42022 (2017). SRCEC3 2045-2322 10.1038/srep42022 28181535 PMC5299514

[9] Hrabe J. Hrabetova S., “Time-resolved integrative optical imaging of diffusion during spreading depression,” Biophys. J. 117(10), 1783–1794 (2019). BIOJAU 0006-3495 10.1016/j.bpj.2019.08.031 31542225 PMC7018988

[10] Zhu P. K. et al., “Nanoscopic visualization of restricted nonvolume cholinergic and monoaminergic transmission with genetically encoded sensors,” Nano Lett. 20(6), 4073–4083 (2020). NALEFD 1530-6984 10.1021/acs.nanolett.9b04877 32396366 PMC7519949

[11] Chen P. et al., “A noninvasive fluorescence imaging-based platform measures 3D anisotropic extracellular diffusion,” Nat. Commun. 12, 1913 (2021). NCAOBW 2041-1723 10.1038/s41467-021-22221-0 33772014 PMC7997923

[12] Xiong H. et al., “Probing neuropeptide volume transmission *in vivo* by a novel all-optical approach,” bioRxiv (2021).

[13] Nicholson C. Hrabětová S., “Brain extracellular space: the final frontier of neuroscience,” Biophys. J. 113(10), 2133–2142 (2017). BIOJAU 0006-3495 10.1016/j.bpj.2017.06.052 28755756 PMC5700249

[r14] Hrabetova S. et al., “Unveiling the extracellular space of the brain: from super-resolved microstructure to *in vivo* function,” J. Neurosci. 38(44), 9355–9363 (2018). JNRSDS 0270-6474 10.1523/JNEUROSCI.1664-18.2018 30381427 PMC6706003

[15] Sykova E. Nicholson C., “Diffusion in brain extracellular space,” Physiol. Rev. 88(4), 1277–1340 (2008). PHREA7 0031-9333 10.1152/physrev.00027.2007 18923183 PMC2785730

[16] Octeau J. C. et al., “Transient, consequential increases in extracellular potassium ions accompany channelrhodopsin2 excitation,” Cell Rep. 27(8), 2249–2261.e7 (2019). 10.1016/j.celrep.2019.04.078 31116972 PMC6582980

[17] Vargová L. Syková E., “Extracellular space diffusion and extrasynaptic transmission,” Physiol. Res. 57(Suppl. 3), S89–S99 (2008). PHRSEJ 0862-8408 10.33549/physiolres.931603 18481911

[18] van den Pol A. N., “Neuropeptide transmission in brain circuits,” Neuron 76(1), 98–115 (2012). NERNET 0896-6273 10.1016/j.neuron.2012.09.014 23040809 PMC3918222

[19] Tønnesen J. Inavalli V. V. G. K. Nägerl U. V., “Super-resolution imaging of the extracellular space in living brain tissue,” Cell 172(5), 1108–1121.e15 (2018). CELLB5 0092-8674 10.1016/j.cell.2018.02.007 29474910

[20] Godin A. G. et al., “Single-nanotube tracking reveals the nanoscale organization of the extracellular space in the live brain,” Nat. Nanotechnol. 12(3), 238–243 (2017). NNAABX 1748-3387 10.1038/nnano.2016.248 27870840

[21] Lau L. W. et al., “Pathophysiology of the brain extracellular matrix: a new target for remyelination,” Nat. Rev. Neurosci. 14(10), 722–729 (2013). NRNAAN 1471-003X 10.1038/nrn3550 23985834

[22] Poole J. J. A. Mostaço-Guidolin L. B., “Optical microscopy and the extracellular matrix structure: a review,” Cells 10(7), 1760 (2021). 10.3390/cells10071760 34359929 PMC8308089

[23] Wolak D. J. Thorne R. G., “Diffusion of macromolecules in the brain: implications for drug delivery,” Mol. Pharm. 10(5), 1492–1504 (2013). 10.1021/mp300495e 23298378 PMC3646902

[r24] Soria F. N. et al., “Current techniques for investigating the brain extracellular space,” Front. Neurosci. 14, 1076 (2020).1662-453X 10.3389/fnins.2020.570750

[25] Liu C. Goel P. Kaeser P. S., “Spatial and temporal scales of dopamine transmission,” Nat. Rev. Neurosci. 22(6), 345–358 (2021). NRNAAN 1471-003X 10.1038/s41583-021-00455-7 33837376 PMC8220193

[26] Nicholson C. Syková E., “Extracellular space structure revealed by diffusion analysis,” Trends Neurosci. 21(5), 207–215 (1998). TNSCDR 0166-2236 10.1016/S0166-2236(98)01261-2 9610885

[27] Stucht D. et al., “Highest resolution in vivo human brain MRI using prospective motion correction,” PLoS One 10(7), e0133921 (2015). POLNCL 1932-6203 10.1371/journal.pone.0133921 26226146 PMC4520483

[28] Nicholson C. Phillips J., “Ion diffusion modified by tortuosity and volume fraction in the extracellular microenvironment of the rat cerebellum,” J. Physiol. 321(1), 225–257 (1981). JPHYA7 0022-3751 10.1113/jphysiol.1981.sp013981 7338810 PMC1249623

[29] Odackal J. et al., “Real-time iontophoresis with tetramethylammonium to quantify volume fraction and tortuosity of brain extracellular space,” J. Vis. Exp. (125), e55755 (2017). 10.3791/55755

[30] Soria F. N. et al., “Synucleinopathy alters nanoscale organization and diffusion in the brain extracellular space through hyaluronan remodeling,” Nat. Commun. 11(1), 3440 (2020). NCAOBW 2041-1723 10.1038/s41467-020-17328-9 32651387 PMC7351768

[31] Biermann B. et al., “Imaging of molecular surface dynamics in brain slices using single-particle tracking,” Nat. Commun. 5, 3024 (2014). NCAOBW 2041-1723 10.1038/ncomms4024 24429796 PMC3905702

[32] Wang Z. G. et al., “Real-time dissecting the dynamics of drug transportation in the live brain,” Nano Lett. 21(1), 642–650 (2021). NALEFD 1530-6984 10.1021/acs.nanolett.0c04216 33290082

[33] McKenna M. et al., “Multiple particle tracking detects changes in brain extracellular matrix and predicts neurodevelopmental age,” ACS Nano 15(5), 8559–8573 (2021). ANCAC3 1936-0851 10.1021/acsnano.1c00394 33969999 PMC8281364

[34] Tao L. Nicholson C., “Diffusion of albumins in rat cortical slices and relevance to volume transmission,” Neuroscience 75(3), 839–847 (1996). 10.1016/0306-4522(96)00303-X 8951877

[35] Thevalingam D. et al., “Brain extracellular space of the naked mole-rat expands and maintains normal diffusion under ischemic conditions,” Brain Res. 1771, 147646 (2021). BRREAP 0006-8993 10.1016/j.brainres.2021.147646 34499876

[36] Veerapathiran S. Wohland T., “Fluorescence techniques in developmental biology,” J. Biosci. 43(3), 541–553 (2018). JOBSDN 0250-5991 10.1007/s12038-018-9768-z 30002271

[37] Binder D. K. et al., “*In vivo* measurement of brain extracellular space diffusion by cortical surface photobleaching,” J. Neurosci. 24(37), 8049–8056 (2004). JNRSDS 0270-6474 10.1523/JNEUROSCI.2294-04.2004 15371505 PMC6729785

[38] Papadopoulos M. C. Binder D. K. Verkman A. S., “Enhanced macromolecular diffusion in brain extracellular space in mouse models of vasogenic edema measured by cortical surface photobleaching,” FASEB J. 19(3), 425–427 (2005). FAJOEC 0892-6638 10.1096/fj.04-2834fje 15596484

[39] Magzoub M. et al., “Extracellular space volume measured by two-color pulsed dye infusion with microfiberoptic fluorescence photodetection,” Biophys. J. 96(6), 2382–2390 (2009). BIOJAU 0006-3495 10.1016/j.bpj.2008.12.3916 19289063 PMC2907719

[40] Dix J. A. Verkman A., “Mapping of fluorescence anisotropy in living cells by ratio imaging. Application to cytoplasmic viscosity,” Biophys. J. 57(2), 231–240 (1990). BIOJAU 0006-3495 10.1016/S0006-3495(90)82526-1 2317548 PMC1280665

[41] Suhling K. et al., “Time-resolved fluorescence anisotropy imaging applied to live cells,” Opt. Lett. 29(6), 584–586 (2004). OPLEDP 0146-9592 10.1364/OL.29.000584 15035478

[42] Zheng K. et al., “Monitoring nanoscale mobility of small molecules in organized brain tissue with time-resolved fluorescence anisotropy imaging,” in Nanoscale Imaging of Synapses, Nägerl U. V. Triller A., Eds., pp. 125–143, Humana Press, New York (2014).

[43] Schuster B. S. et al., “Particle tracking in drug and gene delivery research: state-of-the-art applications and methods,” Adv. Drug Deliv. Rev. 91, 70–91 (2015). ADDREP 0169-409X 10.1016/j.addr.2015.03.017 25858664 PMC4813524

[44] Wang Y. et al., “Localization events-based sample drift correction for localization microscopy with redundant cross-correlation algorithm,” Opt. Express 22(13), 15982–15991 (2014). OPEXFF 1094-4087 10.1364/OE.22.015982 24977854 PMC4162368

[45] Khater I. M. Nabi I. R. Hamarneh G., “A review of super-resolution single-molecule localization microscopy cluster analysis and quantification methods,” Patterns 1(3), 100038 (2020). 10.1016/j.patter.2020.100038 33205106 PMC7660399

[46] Paviolo C. Cognet L., “Near-infrared nanoscopy with carbon-based nanoparticles for the exploration of the brain extracellular space,” Neurobiol. Dis. 153, 105328 (2021). NUDIEM 0969-9961 10.1016/j.nbd.2021.105328 33713842

[47] Welsher K. Sherlock S. P. Dai H., “Deep-tissue anatomical imaging of mice using carbon nanotube fluorophores in the second near-infrared window,” PNAS 108(22), 8943–8948 (2011). 10.1073/pnas.1014501108 21576494 PMC3107273

[48] Gong H. Peng R. Liu Z., “Carbon nanotubes for biomedical imaging: the recent advances,” Adv. Drug Deliv. Rev. 65(15), 1951–1963 (2013). ADDREP 0169-409X 10.1016/j.addr.2013.10.002 24184130

[49] Paviolo C. et al., “Nanoscale exploration of the extracellular space in the live brain by combining single carbon nanotube tracking and super-resolution imaging analysis,” Methods 174, 91–99 (2020). MTHDE9 1046-2023 10.1016/j.ymeth.2019.03.005 30862507

[50] Linarès-Loyez J. et al., “Self-interference (SELFI) microscopy for live super-resolution imaging and single particle tracking in 3D,” Front. Phys. 7, 68 (2019). 10.3389/fphy.2019.00068

[51] Yuan G. et al., “Two mechanisms determine quantum dot blinking,” ACS Nano 12(4), 3397–3405 (2018). ANCAC3 1936-0851 10.1021/acsnano.7b09052 29579376

[52] Nance E. A. et al., “A dense poly (ethylene glycol) coating improves penetration of large polymeric nanoparticles within brain tissue,” Sci. Transl. Med. 4(149), 149ra119–149ra119 (2012). STMCBQ 1946-6234 10.1126/scitranslmed.3003594

[53] Agnati L. F. et al., “Understanding wiring and volume transmission,” Brain Res. Rev. 64(1), 137–159 (2010). BRERD2 0165-0173 10.1016/j.brainresrev.2010.03.003 20347870

[54] Russo A. F., “Overview of neuropeptides: awakening the senses?” Headache 57, 37–46 (2017). HEADAE 0017-8748 10.1111/head.13084 28485842 PMC5424629

[55] Del-Bel E. De-Miguel F. F., “Extrasynaptic neurotransmission mediated by exocytosis and diffusive release of transmitter substances,” Front. Syn. Neurosci. 10, 13 (2018). 10.3389/fnsyn.2018.00013

[56] Sabatini B. L. Tian L., “Imaging neurotransmitter and neuromodulator dynamics *in vivo* with genetically encoded indicators,” Neuron 108(1), 17–32 (2020). NERNET 0896-6273 10.1016/j.neuron.2020.09.036 33058762

[57] Patriarchi T. et al., “Ultrafast neuronal imaging of dopamine dynamics with designed genetically encoded sensors,” Science 360(6396), eaat4422 (2018). SCIEAS 0036-8075 10.1126/science.aat4422 29853555 PMC6287765

[r58] Sun F. et al., “A genetically encoded fluorescent sensor enables rapid and specific detection of dopamine in flies, fish, and mice,” Cell 174(2), 481–496.e19 (2018). CELLB5 0092-8674 10.1016/j.cell.2018.06.042 30007419 PMC6092020

[59] Sun F. et al., “Next-generation GRAB sensors for monitoring dopaminergic activity *in vivo*,” Nat. Methods 17(11), 1156–1166 (2020).1548-7091 10.1038/s41592-020-00981-9 33087905 PMC7648260

[60] Feng J. et al., “A genetically encoded fluorescent sensor for rapid and specific *in vivo* detection of norepinephrine,” Neuron 102(4), 745–761.e8 (2019). NERNET 0896-6273 10.1016/j.neuron.2019.02.037 30922875 PMC6533151

[61] Wan J. et al., “A genetically encoded sensor for measuring serotonin dynamics,” Nat. Neurosci. 24(5), 746–752 (2021). NANEFN 1097-6256 10.1038/s41593-021-00823-7 33821000 PMC8544647

[62] Thorne R. G. Nicholson C., “*In vivo* diffusion analysis with quantum dots and dextrans predicts the width of brain extracellular space,” PNAS 103(14), 5567–5572 (2006). 10.1073/pnas.0509425103 16567637 PMC1459394

